# Tyrosine kinase inhibitor SU11274 increased tumorigenicity and enriched for melanoma-initiating cells by bioenergetic modulation

**DOI:** 10.1186/s12885-016-2341-y

**Published:** 2016-05-12

**Authors:** Lucia Kucerova, Lucia Demkova, Svetlana Skolekova, Roman Bohovic, Miroslava Matuskova

**Affiliations:** Laboratory of Molecular Oncology, Cancer Research Institute of Biomedical Research Centre, Slovak Academy of Sciences, Dubravska cesta 9, Bratislava, 845 05 Slovakia

**Keywords:** Human melanoma, Melanoma-initiating cells, c-Met receptor, SU11274, Bioenergetic modulation

## Abstract

**Background:**

Small molecule inhibitor of tyrosine kinase activity, compound SU11274, was reported to have antitumorigenic and antimetastatic effect in melanoma. In this study, we evaluated, whether similar effect could be achieved also in other melanoma cells including highly tumorigenic and hypermetastatic variant.

**Methods:**

The effect of SU11274 was evaluated in adherent and non-adherent melanosphere cultures of human melanoma cells M14, M4Beu, A375 and EGFP-A375/Rel3. Tumorigenicity of SU11274-treated cells was tested by limiting dilution assay in xenograft model in vivo.

**Results:**

Here we show that SU11274 enriched for melanoma-initiating cells in vivo. SU11274 substantially decreased number of cells in adherent and spheroid cultures, but increased their tumorigenic potential as determined by higher frequency of tumor-initiating cells in vivo. SU11274 treatment was not associated with any significant alteration in the expression of stem cell markers, but the inhibitor stimulated higher level of pluripotent markers. SU11274-treated melanoma cells exhibited higher ATP content and lactate release indicative of increased glycolysis. Our data suggest that the SU11274 altered bioenergetic state of the cells. Indeed, pharmacological intervention with a glycolytic inhibitor dichloroacetate significantly reduced SU11274-promoted increase in melanoma-initiating cells and decreased their tumorigenicity.

**Conclusions:**

Our data suggest critical role of glycolysis regulation in melanoma-initiating cells. Moreover, these data unravel substantial plasticity of melanoma cells and their adoptive mechanisms, which result in ambivalent response to therapeutic targeting.

## Background

Small molecule inhibitor SU11274 was initially developed to specifically inhibit c-Met receptor signaling [1]. Receptor tyrosine kinase c-Met is a receptor for hepatocyte growth factor/scatter factor (HGF), pleiotropic cytokine controlling pro-migratory, anti-apoptotic and mitogenic signals [2]. c-Met activation evokes biological responses, globally referred to as ‘invasive growth’, thus being potential therapeutic target in metastatic cancer [3]. Promise of anti-c-Met drugs is based on their activity on multiple stages of cancer development, from initiation through progression to metastasis [4]. Moreover, the inappropriate c-Met signaling occurs in virtually all types of solid tumors [5]. In our previous study we have confirmed high expression of the c-Met receptor in a model cell line EGFP-A375/Rel3 derived as hypermetastatic and highly tumorigenic variant of human melanoma cell line A375 [6]. Our experiments in the above mentioned study have shown antiproliferative effect of SU11274 in vitro, but tumor supporting effect in vivo (see Fig. 6), when used as an augmentation to support antitumor effect of gene therapy-based approach [6]. It has been reported that the intraperitoneal administration of SU11274 achieved significant inhibitory effect on liver metastasis induced by the intrasplenic injection of human metastatic melanoma cells the HT168-M1 [7]. Moreover, the intratumor injection of SU11274 had high efficacy in vivo and this treatment reduced tumor volume by 7-fold as compared with control tumors induced by RU-P melanoma cells [8]. Based on the findings we wanted to explore the effect of SU11274 on various melanoma cell lines including our model of hypermetastatic variant, which was not examined so far.

Bulk tumor comprises subpopulations of non-tumorigenic and tumorigenic cells, which can reversibly transit among their states (tumorigenic stem cells vs. non-tumorigenic cells) [9]. Tumor-initiating cells (tumorigenic or so-called cancer stem cells) give rise to tumors in transplantation assays in vivo and they were associated with specific surface markers in human melanoma [10, 11]. Experiments suggested significant level of plasticity in melanoma cells and many markers were reversibly turned on and off in a manner that did not correlate with the ability to form a tumor [12–14]. Growth of tumor cells in three-dimensional multicellular tumor spheroid cultures enables to maintain their tumorigenic potential [15–17]; and therefore we suggested to use it to explore the effect of SU11274 treatment on tumor initiating potential of melanoma cells. It was previously shown that decreased tumor sphere formation by the inhibition of c-Met correlated with preventing metastatic disease and inhibiting stem cell function in pancreatic carcinoma [18].

There is not much known about the metabolic regulation of cancer stem cell function, but bioenergetic modulation was shown to counteract stem cell features and sensitized cells to kinase inhibitors [19–22]. Bioenergetic modulators could be actually used in the antitumor treatment [21, 23, 24]; but recent evidence suggested specific metabolic behavior of melanoma cells [25, 26]. These reports prompted us to test potential of bioenergetic modulation to interfere with the tumor initiation in melanoma.

We evaluated the effect of SU11274 inhibitor in both adherent and spheroid melanoma cultures in vitro; and the effect on tumor growth and initiation in vivo. Our data show that SU11274-treated cells were enriched for melanoma-initiating cells; they had significantly increased tumorigenic potential. This effect could be counteracted by bioenergetic modulation with a glycolytic inhibitor dichloroacetate (DCA).

## Methods

### Chemicals

Following reagents were purchased from Sigma-Aldrich (St. Louis, MO): SU11274, dacarbazine (DACA), dichloroacetate (DCA), 3-bromo-pyruvate (3BrPA). Crizotinib (Pfizer Inc., Mission, KS) was kindly provided by the National Cancer Institute, Bratislava.

### Cells and cell lines

Human malignant melanoma cell lines A375 (ATCC® CRL-1619™), M14 and M4Beu [27] (kindly provided by Dr. Bizik, CRI BMC SAS Bratislava) were propagated in DMEM medium supplemented with 5 % of fetal bovine serum, glutamine, penicillin/streptomycin and amphotericin. Cell lines EGFP-A375 and EGFP-A375/Rel3 were derived as described [6, 28]. EGFP-A375/Rel3 cells will be designated Rel3 (3rd relapse) for the rest of the study. These cells were derived by expansion of tertiary relapse of tumors after in vivo treatment with prodrug converting cell-based gene/prodrug therapy approach.

Melanosphere culture was performed in ultra-low adherent plates and serum-free medium supplemented with B27, EGF and bFGF as described in detail elsewhere [29]. Usually 5–10,000 melanoma cells per 3 ml culture media per well was cultured in 6-well plates for 7 days. Melanospheres were collected by centrifugation, dissociated by trypsinization, viable cell count was determined by trypan blue exclusion assay and single cell suspension was used for further cultivation or treatments. In order to assess cellular morphology, cell or spheroid images were taken by the IncuCyte ZOOM™ Kinetic Imaging System (Essen BioScience, Welvyn Graden City, UK). Cell confluence was calculated by the IncuCyte ZOOM software 2012A.

### ATP and cell viability assay

Relative ATP content per cell was determined by the CellTiter-Glo™ Luminescent Cell Viability Assay (Promega Corporation, Madison, WI). Cells were counted using trypan blue exclusion assay, 50–100 μl cell suspension was mixed with equal volume of the luminescent reagent and luminescence in relative luminescent units (RLU) was determined on the LumiStar GALAXY reader (BMG Labtechnologies, Offenburg, Germany).

Relative viability of the cells was measured by the same method. In the adherent conditions, cells were plated at 1500–2000 cells/100 μl media per well in 96-well white-walled plates, let to adhere for 24 h, supplemented with the drug(s) to reach indicated final concentration and treated for 3–5 days. In the melanosphere conditions, 500 cells/100 μl media per well in 96-well ultra-low attachment plates were supplemented with the indicated compounds and treated for 5–7 days. At the end of the melanosphere experiment, luminescent reagent was added to the wells (ratio 1:1), incubated for 15 min at room temperature, lysate transferred to the white walled 96-well plates and a relative luminescence was measured as above. Experiments were performed in quadruplicates at least four times with similar results and the representative result is shown, the average relative luminescence of the cells without any treatment was set to 100 % and calculated relative values were expressed as means + SD.

### Chemiluminescent BrdU cell proliferation ELISA

Triplicates of 3000 Rel3 cells per well were seeded in 96-well black-walled plates 24 h prior to the treatment start. Cells were treated with the indicated concentration of SU11274 for 6 days. BrdU was added to the wells 18 h prior to evaluation. Time and the drug doses were chosen based on preliminary experiments (not shown). BrdU incorporation was determined by the Cell Proliferation ELISA, BrdU (chemiluminescent, Roche Diagnostics, Mannheim, Germany) on the LUMIstar GALAXY reader (BMG Labtechnologies, Offenburg, Germany). BrdU incorporation of the cells incubated without any treatment was taken as 100 % by default. Values were expressed as an average of relative BrdU incorporation + SD. Experiments were repeated twice with similar results and a representative outcome is shown.

### Glucose uptake and lactate release assay

Measurement of the glucose uptake was done with the Glucose Uptake Colorimetric Assay Kit (BioVision Inc., Milpitas, CA). Melanoma cells were treated for 7 days with 1 μM SU11274 inhibitor, counted and resuspended to obtain 500,000 cells per 0.5 ml media. 2-deoxyglucose was added to the suspension and a measurement proceeded according to the manufacturer’s protocol. Lactate release from the treated cells was determined by Lactate Colorimetric Assay Kit II (BioVision Inc., Milpitas, CA). Treated cells were seeded at a density of 500,000 cells/well/0,5 ml media in 24-well plates for 16 h. Medium was discarded after incubation, cells were lysed in the reaction mix and the analysis proceeded as recommended in the manufacturer’s protocol. Values were determined on xMark™ Microplate Absorbance Spectrophotometer (Bio-Rad Laboratories, Hercules, CA). Experiment was repeated at least twice, each value determined in triplicates and a representative outcome is shown.

### Flow cytometry

For the detection of the expression of surface markers, anti-human c-Met-PE (Sino Biological Inc. Beijing, China) antibodies were used. Dead cells were excluded based on the DAPI (4′, 6-diamidino-2-phenylindole) staining. Cells were analyzed using BD Canto II cytometer (Beckton Dickinson, Franklin Lakes, NJ) equipped with FACSDiva program. FCS Express software was used for the evaluation.

### Protein arrays and analysis

Proteome profile of the melanosphere cells cultured for 7 days in the presence of 1 μM SU11274 was evaluated by the Proteome Profiler™ Human Phospho-Kinase Antibody Array and the Human Pluripotent Stem Cell Antibody Array (R&D Systems™ Inc., Minneapolis MN). Cells were dissociated, counted and lysed in a lysis buffer at a concentration of 10^7^ per ml. Protease inhibitors were added to the lysis buffer for the pluripotent stem cell array at recommended concentration (Complete Protease Inhibitor Cocktail Tablets, Roche Diagnostics Deutschland GmbH, Mannheim, Germany). Protein lysate (350 μg total protein) was loaded on the membranes with blotted antibodies and evaluated as recommended by the manufacturer.

Phoshorylation status of c-Met was analyzed by western blotting. Cells were lysed in buffer containing 50 mM Tris HCl (pH 7.4), 1 % NP40, 0,5 % SDS, 150 mM NaCl, 2 mM EDTA, 50 mM NaF, 0,2 mM sodium ortho-vanadate (Na3VO4), and protease inhibitor cocktail tablets Complete (Roche, cat. no. 04 693 116 001). Lysates were prepared for SDS-PAGE by adding 10 μg of protein to 4× Laemelli’s loading buffer (Bio-Rad, cat. no. 161-0747). Samples were denatured at 95 °C for 4 min and centrifuged for 30 s at 5000 rpm, prior to electrophoresis. Protein samples were loaded onto a 10 % polyacrylamide gel (Bio-Rad, TGX Stain-Free FastCast Acrylamide Kit, 10 %, Cat. No. 161-0183) and electrophoresed for 1 h at 200 V in a Mini-Protean Tetra Cell (Bio-Rad, Cat. No.165-8004) using 10× Tris/Glycine/SDS Running buffer pH 8.3 (Bio-Rad, cat. no. 161-0732). Proteins were transferred onto a nitrocellulose membrane using the Mini Trans-Blot Cell Module (Bio-Rad, cat. no. 1703811) in a transfer buffer (10× Tris/Glycine/SDS Buffer pH 8.3 and 20 % methanol) at 100 V for 1 h. Specific phospho-Met antibody (Tyr1349, 130H2, rabbit mAb, Cell Signaling Technologies, cat. no. 3133) was used, monoclonal Anti-β-actin (SIGMA-ALDRICH, cat. no. A1978) served as a loading control. Immunoblots were visualized using enhanced chemiluminescence (Bio-Rad, Clarity Western ECL Substrate, cat. no. 170-5060).

### In vivo animal studies

Project was performed in the approved animal facility (licence number SK PC 14011) as approved by the institutional ethic committee and by the national competence authority (State Veterinary and Food Administration of the Slovak Republic, registration number Ro 3108/14-221) in compliance with the Directive 2010/63/EU of the European Parliament and the European Council and the Regulation 377/2012 on the protection of animals used for scientific purposes. Six weeks-old athymic nude mice (Balb/c-nu/nu) were used in accordance with the institutional guidelines under the approved protocols. It was determined in the preliminary studies that the 10^6^ of EGFP-A375 cells exhibited 100 % tumor penetrance when injected s.c. in a serum-free medium. In order to compare the tumorigenicity of melanoma cells, gradually decreasing numbers of EGFP-A375 and Rel3 cells were injected s.c. in the 100 μl serum-free media. The frequency of the tumor-initiating cells was determined by the extreme limiting dilution analysis (ELDA) [30]. To evaluate the effect of SU11274 inhibitor on the tumor-initiating capabilities, melanoma cells were treated with 1 μM SU11274 for 7 days in vitro and these cells were injected 2 × 10^5^/100 μl/mouse s.c. Tumor take rates for the melanosphere cells was determined as a proportion of tumors growing to all inoculations of given cell number injected in the 1:1 serum free-DMEM diluted matrigel (ECM Gel from Engelbreth-Holm-Swarm murine sarcoma, Sigma-Aldrich). Both SU11274 treated and untreated cells in vitro were used to compare the tumor initiating potential. In a synthetic lethality study, the cells from the spheroid cultures in the presence of 1 μM SU11274 were treated for 24 h with the 5 mM DCA, 3.5 μM 3BrPA or 100 μM dacarbazine (DACA) added on the day 4. Cells were let to recover for 48 h in the presence of inhibitor SU11274. Cells were collected by centrifugation, trypsinized, counted and 20,000/site in 1:1 diluted ECM gel injected s.c. to determine their potential for a tumor initiation. The ATP level per well was determined as above in the single cell suspension.

The animals were regularly inspected for the tumor incidence and considered tumor-free when no palpable rigid structure exceeding 1 mm^3^ could be detected. Growing tumors were measured by the caliper and a volume of tumor was calculated according to the formula volume = length × width^2^/2. Results were evaluated as median volume + (min, max). Animals were sacrificed, when the tumors exceeded 1 cm^3^ in accordance with the ethical guidelines or at the experiment endpoint. Animals were designated tumor-free at the experiment endpoint, when no tumor growth was detectable at necropsy.

### Statistical analysis

The Student’s two-sample *t*-test was used for hypothesis testing for the difference in means of two samples, assuming that both samples come from a normal distribution with the standard deviations unknown but assumed equal. The Mann-Whitney *U* test was used to perform a two-sided test of the hypothesis that two independent samples come from distributions with equal medians. The *p*-values with *p* < 0.05 were considered to be statistically significant.

## Results

**Fig. 1:**
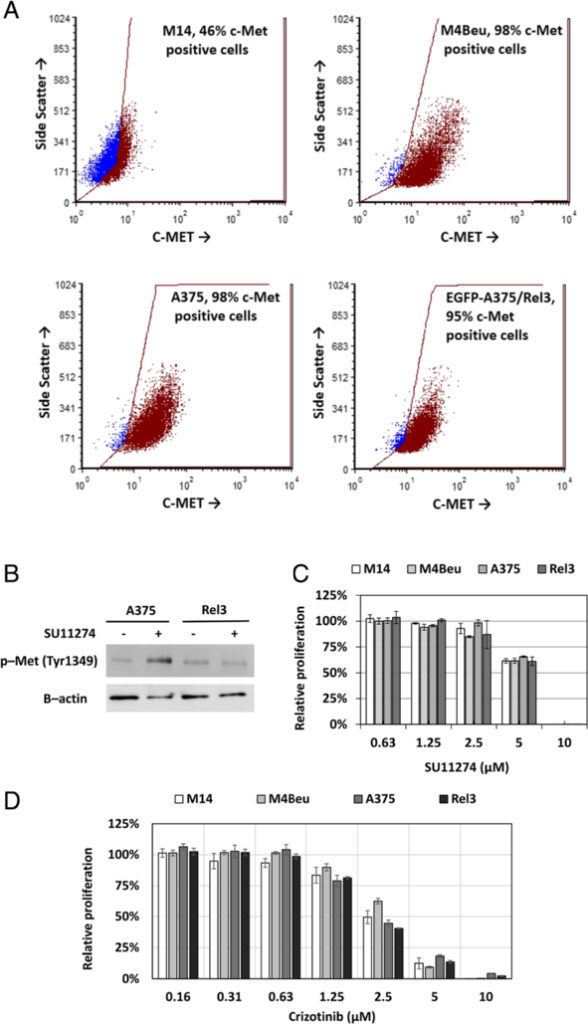
SU11274 and crizotinib inhibit melanoma cell proliferation. **a** Flow cytometry analysis of four different human melanoma cell lines confirmed high expression of c-Met receptor. Anti-human c-Met-PE (Met, HGFR) antibody was used to detect positive cells, DAPI was used as a viability counterstain. **b** Phosphorylation status of c-Met was examined by western blot. Tyr1349 is phosphorylated in A375 and Rel3, SU11274 further increased phosphorylation in A375. **c**–**d** Adherent melanoma cells exhibited similar IC_50_ values for c-Met inhibitors SU11274 or crizotinib. Relative viability was determined by luminescent viability assay on day 3. Values were calculated from the quadruplicates as means + SD

**Fig. 2:**
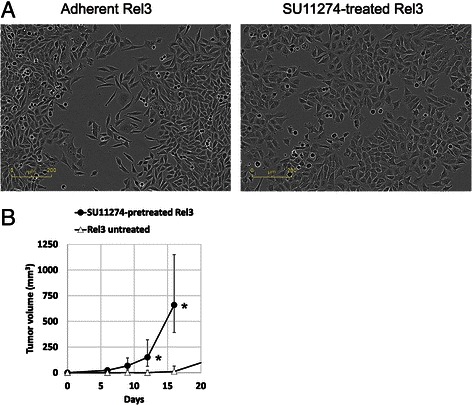
Treatment with SU11274 alters morphology of melanoma cells and their tumorigenicity. **a** Treatment with 1 μM SU11274 induces inhibition of cell proliferation and morphological alteration in Rel3 cells. Colonies of SU11274-treated cells appeared less scattered and formed tighter intercellular junctions. Scale bar 200 μm. **b** Two × 10^5^ of untreated or SU11274-treated Rel3 cells (1 μM SU11274 for 7 days in vitro) were injected subcutaneously into the flank of immunodeficient mice. Tumor burden was significantly higher in xenografts from SU11274-treated cells, data show median tumor volume with vertical bars depicting maximum and minimum tumor volume in group, *p ≤ 0.05

Recently we described novel hypermetastatic human melanoma cell line EGFP-A375/Rel3 (designated Rel3 in the following text) [6]. It was derived from parental EGFP-A375 [28] by three rounds of consecutive in vivo passaging as the third relapse which could regrow after the experimental therapy with prodrug-converting mesenchymal stromal cells. Rel3 cell line is highly tumorigenic and produces massive lung colonization upon intravenous injection indicative of its aggressiveness. We decided to examine the antitumor potential of SU11274 in malignant melanoma cell lines M14, M4Beu [27], A375 [28] and Rel3 cells (derived hypermetastatic variant of A375) [6]. We confirmed high level of the c-Met receptor on cell surface by flow cytometry in these cells. The c-Met expression was detected on the 46 % of M14 cells, 97.7 % of M4Beu cells, 98.0 % of A375 cells and 95.2 % of Rel3 cells (Fig. 1a). SU11274 can inhibit HGF-stimulated phosphorylation of c-Met on Tyr1234/1235 [31]. C-Met is not phosphorylated on these Tyr residues in A375 [32]. However, our data have shown that SU11274 increased phosphorylation of c-Met on Tyr1349, which was phosphorylated in both A375 and Rel3 cells (Fig. 1b). In addition to SU11274 as a selective c-Met inhibitor with the IC_50_ of 10 nM in a cell-free assay, Crizotinib (PF-02341066) as another ATP-competitive small-molecule inhibitor of the catalytic activity of c-Met the IC_50_ of 11 nM and in cell-free assay was used [33, 34]. Examined cell lines exhibited very similar IC_50_ for these two different c-Met inhibitors SU11274 ranging 4–5 μM (Fig. 1c) and crizotinib ranging 1.25–3 μM (Fig. 1d) in standard adherent cultures. SU11274 treatment caused alteration in cellular morphology from narrow spindle shape to flatter rounded morphology and less scattered colonies (Fig. 2a). SU11274-treated cells were subsequently injected as xenografts and they were more tumorigenic in vivo (Fig. 2b). When we injected 5 × 10^5^ cells, the median tumor volume was significantly higher in the SU11274-pretreated group - 721.2 mm^3^ versus 395.8 mm^3^ in control group by day 16). When 2 × 10^5^ cells were injected, tumor take rate was also higher in the SU11274-pretreated group with 4 out of 4 injected tumors growing in contrast to the untreated group, where only 2 out of 4 inoculates grew with a longer dormancy period. Tumor growth kinetic is shown in (Fig. 2b).

**Fig. 3:**
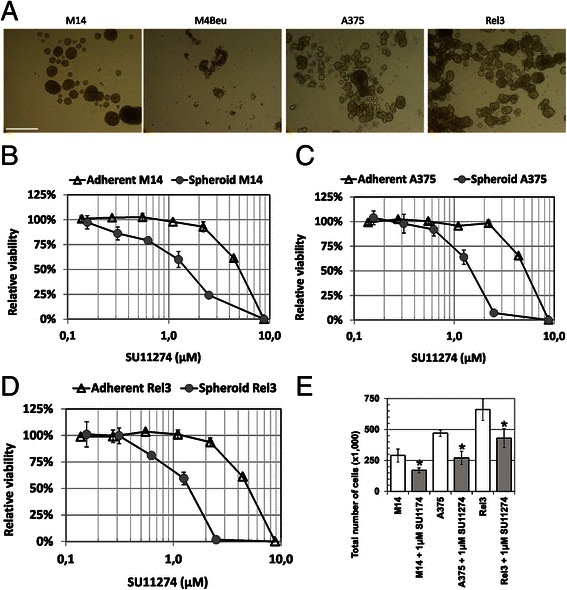
Melanosphere propagation increases tumor cell sensitivity to SU11274. **a** Human melanoma cells were seeded into ultra-low attachment plates in serum-free DMEM/F12 medium supplemented with B27, EGF and bFGF. M14, EGFP-A375 and Rel3 could be propagated and formed tight melanospheres. Cell line M4Beu did not form spheroids and did not proliferate under these culture conditions. Scale bar 500 μm. **b**–**d** Sensitivity of the adherent versus spheroid cultures to SU11274 was compared. Non-adherent melanoma cells M14, A375 and Rel3 were more sensitive to SU11274 inhibitor in comparison to adherent cells. Relative viability was determined by luminescent ATP-based viability assay after 5-day treatment. Values were calculated from the quadruplicates as means + SD. **e** Spheroid cultures were initiated from the 5000 cells seeded per well in 6-well plates with or without 1 μM SU11274. Total number of cells per well was counted 7 days later. SU11274-treatment significantly reduced a number of cells in comparison to untreated controls, *p < 0.05

In order to examine the effect of SU11274 on melanoma-initiating cells we switched cells to non-adherent melanosphere cultures [35, 36]. M4Beu cells were not able to proliferate under these conditions and did not form melanospheres (Fig. 3a). M14, A375 and Rel3 cells propagated and expanded in spheroid conditions (at least for more than 10 consecutive passages). Direct comparison of adherent and non-adherent cultures unraveled increased sensitivity to SU11274 in melanospheres (Fig. 3b–d). SU11274 also significantly inhibited cell proliferation. There were 6.5 × 10^5^ Rel3 cells in control versus 4.2 × 10^5^ Rel3 cells after SU11274 treatment, which is a 35 % inhibition of the proliferation. (Fig. 2e). The effect of SU11274 on tumor initiation frequencies was evaluated by extreme limiting dilution assay (ELDA) [30]. We injected gradually decreasing number of the cells after adherent and melanosphere culture and determined a proportion of growing tumors. For adherent parental A375 cells, frequency of tumor initiating cells was one in 8.5 × 10^5^. Frequency of tumor initiating cells in Rel3 was one in 2.4 × 10^5^, which was 3.5 higher corresponding to increased tumorigenicity. More importantly, melanosphere cultures further increased the frequency of tumor initiating cells to 1 out of 3.3 × 10^4^ spheroid Rel3 cells (7-fold increase in comparison to adherent culture). Tumor-initiating cell frequencies in SU11274-treated cells was determined to be 1 out of 3289 cells (*p* value 1 × 10^−5^), which was a 10-fold enrichment for tumor-initiating cells by SU11274. Same effect was achieved in M14 cells, where stem cell frequencies determined in vivo were 1 out of 3.8 × 10^4^ spheroid M14 cells in contrast to 1 out of 1.0 × 10^3^ SU11274-treated spheroid M14 cells. This represents a 4-fold enrichment in tumor initiating cell frequency (Table 1).

**Table 1:** Frequency of tumor initiating cells

Confidence intervals for 1/(stem cell frequency)			
Rel3	Lower	Estimate	Upper
Spheroid cells	84,848	33,233	13,016
SU11274-treated spheroid cells	7208	3289	1501
M14	Lower	Estimate	Upper
Spheroid cells	168,352	38,964	9018
SU11274-treated spheroid cells	29,964	10,211	3479

Human melanoma cells were cultured in non-adherent conditions for 7 days in the presence or absence of 1 μM SU11274. Cells were counted, diluted in 1:1 serum-free medium: ECM gel and injected s.c. (*n* = 4/each group). Following numbers were injected: 50,000; 10,000; 5000; 1000; 500; 100; 50 and 10 Rel3 cells, and 20,000; 2000; 200 and 20 M14 cells based on preliminary test of tumorigenicity. Stem cell frequency was calculated by ELDA analysis, *p* value was ≤ 10^−5^ for the Rel3 cells, and ≤ 0.05 for the M14 cells. The tumor take rate was significantly higher in the SU11274-treated cells: 3 out of 4 inoculations of 500 SU11274-treated EGFP-A375/Rel3 cells gave tumors in contrast to 0 out of 4 inoculations of the untreated cells. Similarly, 4 out of 4 inoculations of 2000 SU11274-treated M14 cells gave tumors in contrast to 0 out of 4 inoculations of the untreated M14 cells

**Fig. 4:**
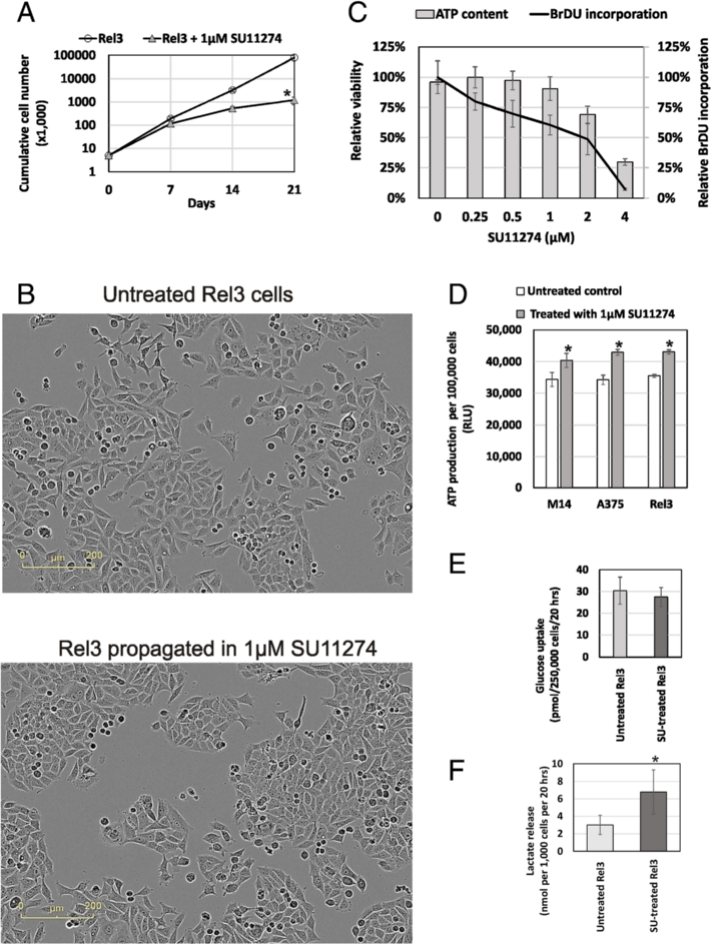
SU112747 mediated bioenergetic alterations. **a** Melanoma cells Rel3 were serially passaged in spheroid culture conditions. Cumulative cell numbers were counted from the number of expanded cells and the inoculum used for each passage. There was a significant difference between the number of cells in SU11274-treated versus untreated cultures with a substantial inhibition of the cell proliferation with 1 μM SU11274. Melanoma cells could be serially propagated the presence of inhibitor (≥10 passages), thus demonstrating that SU11274 does not compromise long-term proliferation potential in vitro. **b** Rel3 cells from the spheroid cultures were trypsinized and plated in adherent culture conditions. Long-term propagation with or without SU11274 shifted cellular morphology from irregular spiked shape to flatter cobblestone morphology and less scattered colonies. **c** Two independent methods were compared to evaluate effect of the treatment with 1 μM SU11274 for 6 days on cell proliferation. Luminescent ATP-based assay has shown 9.4 % inhibition only in contrast to the 39.5 % inhibition of proliferation as determined by relative BrdU incorporation assay. **d** M14, EGFP-A375 and Rel3 cells were treated with 1 μM SU11274 in spheroid conditions for 7 days. Spheroids were trypsinized, viable cell counts determined, cell suspension was mixed with the luminescent reagent from the Luminescent ATP-based Assay and relative ATP-production per 100,000 cells was calculated at least in quadruplicates. SU11274 treatment significantly increased the relative ATP-content per cell in comparison to untreated controls. **e**–**f** Same treatments as in Fig. 4d. were used to determine glucose uptake and lactate release by colorimetric method. Values were expressed as mean + SD, there was no significant difference in glucose uptake between the SU11274-treated and untreated cells. SU11274-treated cells exhibited significantly higher levels of released lactate in comparison to untreated cells, *p < 0.05

Next, we evaluated a long-term serial propagation of cells in the non-adherent conditions with or without SU11274. Rel3 cells could be long-term propagated, although the cumulative cell numbers differed significantly due to the antiproliferative action of the inhibitor (Fig. 4a). Cells from melanospheres were viable; they adhered and proliferated after switching to adherent conditions. Cell morphology after spheroid culture remained similar to morphology of adherent cultures in the presence or absence of SU11274 shifted from irregular spiked shape to flatter cobblestone morphology (Fig. 4b). Obvious discrepancy between minor decrease in the viability and severe decrease in the cell numbers mediated by SU11274 was further examined by BrdU incorporation assay. DNA synthesis and cell cycle progression was substantially more inhibited in comparison to the decrease of ATP level measured by relative viability assay (Fig. 4c). Relative ATP-content per 100,000 cells was significantly higher in cells propagated in SU11274 (Fig. 4d). Further analysis confirmed no significant difference in the glucose uptake, but higher lactate release from the SU11274-treated cells, indicative of their higher dependence on (or a metabolic switch to) aerobic glycolysis (Fig. 4e and f). No effect on ATP levels/cells and tumorigenicity was be observed with crizotinib (data not shown).

**Fig. 5:**
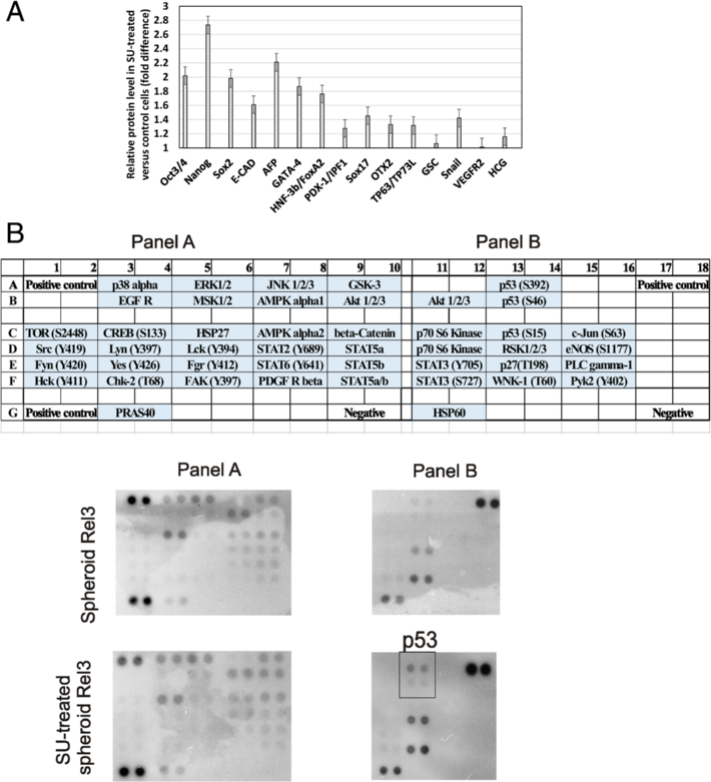
Bioenergetic modulation counteracts the protumorigenic action of the SU11274. **a**–**b** Protein lysates were prepared from untreated controls and Rel3 cells treated with 1 μM SU11274 in spheroid conditions for 7 days. Pluripotent stem cell array has shown expression of all pluripotency markers in Rel3 cells expanded as spheroids. SU11274 treatment further increased level of proteins associated with pluripotency in SU11274-treated cells correlating with their higher tumor initiating properties. Phosphokinase assay screening has shown several active intracellular signaling cascades. Phosphorylated forms of p53 (S392 and S46) were detected in the SU11274-treated cells (lower panel B), which correlates with SU11274 mediated inhibition of cell proliferation. RSK1/2/3 (S380) phosphorylation was increased in SU11274-treated cells. We detected phosphorylation of the following target kinases: ERK1/2 (T202/Y204, T185/Y187), P-RAS40 (T246), Akt 1/2/3 (S473), p38 alpha (T180/Y182), AMPK alpha1 (T183), CREB (S133), GSK-3 alpha/beta(S21/S9), WNK-1 (T60) and HSP60 in both treated and untreated cells

Next, we examined alterations induced by SU11274 on pluripotent stem cell proteins and phosphokinase proteome profile. Melanoma cells express many proteins associated with pluripotency, but SU11274-treated spheroids have increased levels in comparison to the untreated ones (Fig. 5a). Higher level of these transcription factors correlates with increased capability of the treated cells to induce tumor growth. Phosphokinase proteome array demonstrated that SU11274 activated p53 (Fig. 5b, lower panel b), which correlates with inhibition of the cell proliferation shown in Fig. 4a and c. RSK1/2/3 phosphorylation was increased after SU11274 exposure. We detected phosphorylation of following target kinases in Rel3 cells: ERK1/2, p-RAS40, Akt 1/2/3, p38 alpha, AMPK alpha1, CREB, GSK-3 alpha/beta, WNK-1 and HSP60 in both treated and untreated cells.

**Fig. 6:**
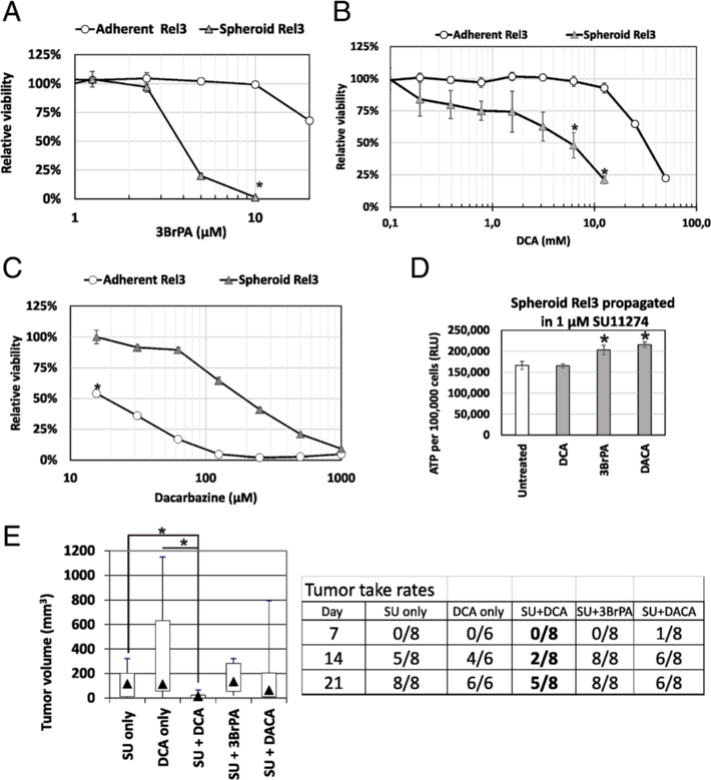
SU11274-mediated increase of tumor initiation can be reverted by bioenergetics odulation ith dichloroacetate. **a**–**c** Sensitivity to glycolytic modulators 3-BrPA, DCA and dacarbazine in adherent and spheroid cultures was compared. Spheroid Rel3 are significantly more sensitive to 3BrPA and DCA, significantly more resistant to dacarbazine. Relative viability was determined by luminescent ATP-based assay. Values were calculated from quadruplicates as means + SD, *p < 0.05. **d**–**e** Rel3 cells were treated with SU11274 in spheroid conditions for 7 days. Following drugs were added for 24 h on day 4 after treatment start: 5 mM DCA, 3.5 μM 3BrPA or 100 μM dacarbazine (DACA). Cells were let to recover for next 48 h in the presence of inhibitor SU11274 only. Spheroids were trypsinized, viable cell counts determined, ATP per cell determined by bioluminescent assay. Then, 1 × 10^4^ cells were mixed with the ECM Gel diluted in serum-free medium and injected s.c. DCA did not change SU11274-mediated increase of the ATP-level per cell, 3BrPA and dacarbazine further increased the ATP level. DCA treatment alone in Rel3 cells did not significantly affect tumor growth or initiation. DCA treatment delayed the tumor onset in SU11274 treated cells and significantly inhibited tumor growth as determined by day 21, *p < 0.05

Based on the previous data suggesting involvement of bioenergetic modulation in SU11274-treated Rel3 cells, we decided to test the ability of bioenergetic modulators to suppress increased tumorigenicity of SU11274-treated cells. Spheroid non-adherent cells were hypersensitive to bioenergetic modulators dichloroacetate (DCA, inhibitor of pyruvate dehydrogenase kinase [23]) and 3-bromopyruvate (3BrPA, a hexokinase and GAPDH inhibitor [24]) in comparison to the adherent cells (Fig. 6a and b). Switch to melanosphere cultures leads to higher cellular dependence on the aerobic glycolysis, which correlates with increased tumor initiating properties of melanosphere cells. Contrastingly, chemotherapeutic alkylating agent dacarbazine, which was clinically approved for the treatment of malignant melanoma [37], was significantly less toxic to spheroid cells in comparison to adherent cells. This demonstrated inherent drug resistance in cultures enriched for melanoma-initiating cells (Fig. 6c). Finally, tumor take rate of the cells treated with SU11274 combined with selected compounds was examined by previously published approach [38]. We expected that in vitro pretreatment targeting tumor-initiating cells would eradicate these from culture and lessen the tumorigenicity. Therefore, SU11274-stimulated cells were co-treated with 3BrPA, DCA or dacarbazine at the IC_50_ for 24 h. Dacarbazine was previously shown to spare the tumor initiating cells; thus, it was used in this assay as a control. Cells were cultured for next 48 h to allow the cell death to occur and viable cells to recover. Combination treatment with DCA did not significantly change SU1174-mediated increase of the ATP content per cell. Compounds 3BrPA or dacarbazine further increased relative ATP level (Fig. 6d). However, DCA decreased tumorigenicity of SU11274-treated cells in vivo, which was not the case for SU11274 combination with 3BrPA or dacarbazine (Fig. 6f). Median tumor volume in the SU11274-treated group was 115.5 mm^3^ in contrast to 14.5 mm^3^ in the DCA pulsed SU11274-treated cells. Moreover, three out of the eight animals did not develop any tumor in contrast to the eight out of the eight in the SU11274 group. DCA treatment alone did not significantly change tumor take rates or median tumor volumes. Our data show that antimelanoma chemotherapeutic drug dacarbazine similarly to bioenergetics modulator 3BrPA does not affect tumor initiating cell subpopulation in Rel3 cells. Combination of the DCA with SU11274 also did not completely eradicate tumor initiation capabilities. We attribute this outcome to the fact that melanoma cells derived from A375 cells harbor mutated B-Raf (V600E), thus tumor initiation capabilities without targeted inhibition of hyperactivated oncogenic pathway were retained. SU11274 compound does not interfere with this signaling axis.

## Discussion

In search for an agent to prevent both growth and metastatic dissemination of melanoma cells we hypothesized that the c-Met receptor was a suitable target [3]. However, experimental evidence suggested that c-Met receptor plays a dual role in oncogenesis. (i) In the mutated, amplified or otherwise genetically altered form, c-Met generates and maintains transformed phenotype, and drives clonal evolution; (ii) in the wild-type form, c-Met contributes to maintain - in the cancer stem cell - the phenotype ‘inherent’ in the stem/progenitor cell of origin [4]. Extensive redundancy of the receptor-tyrosine kinase signaling in cancer cells and receptor cross-talk suggested that there might be inherent or acquired resistance mediated by other signaling cascades compensating for inhibitory effect of the particular small-molecule inhibitor [39]. We detected high surface expression of c-Met receptor in tested melanoma cell lines (Fig. 1a). These cells did not produce detectable HGF into cell culture medium thus excluding c-Met autocrine stimulation (data not shown). We focused predominantly on potential role of the c-Met inhibitor SU11274 in highly metastatic aggressive variant Rel3 [6], as it was suggested as efficient atimelanoma agent [7, 8]. Antiproliferative activity of small molecule inhibitor SU11274 in vitro, unexpectedly, was in contrast to its protumorigenic effect on the Rel3 cells in vivo [6]. More importantly, SU11274 significantly increased frequency of tumor initiating cells. We hypothesize that there might be several reasons for the protumorigenic outcome including individual response of given model cell line, different route of administration or experimental setup. Although intraperitoneal administration of SU11274 decreased metastatic burden in liver of intrasplenically injected mice in orthotopic model [7], the same route of administration supported tumor growth of subcutaneously xenografted melanoma cells Rel3 in heterotopic model (Fig. 2b). Etnyre et al. [8] achieved antitumor effect by direct intratumor injection of compound in model melanoma. Taken together these data stress extreme plasticity of melanoma cells and context-dependent nature between protumorigenic and antitumorigenic action of small molecule inhibitor.

Hierarchical organization of melanoma remains a matter of a debate. It was shown that melanoma cells possess considerable plasticity and represent a tumor type with shallow if any hierarchy [16]. Our data support this notion as we detected high expression of pluripotent proteins in unaffected Rel3 cells. Switching melanoma cells to spheroid non-adherent culture conditions further enriched for melanoma-initiating cells as confirmed by the in vivo assay. We were able to propagate the melanospheres long-term both with or without SU11274 (more than 10 passages corresponding to more than 10 weeks) thus demonstrating the presence of self-renewing cells in vitro and no detrimental effect of the SU11274 on them. We have examined surface marker expression with a particular focus on putative melanoma cancer stem cell markers [10]. We could not find any significant alteration (up- or down-regulation) in any of these markers tested such as c-Kit, CD271, CD133, ABCB5, ABCB1, ALDH1 in the SU11274-treated cells versus the untreated ones (data not shown). Non-adherent culture conditions did not alter melanoma differentiation marker CD146 (M-CAM). It also did not change expression of VEGFR2, VE-cadherin CD144 or angiogenic marker CD31. Based on the data we excluded that increased tumor initiation could be due to a vasculogenic mimicry [40].

SU11274 was previously shown to alter expression profile of the treated cells attributed to its off-target action. Thirty-nine genes belonging to the apoptosis/necrosis, inflammation, oxidative/metabolic stress, heat shock, proliferation/carcinogenesis and growth arrest/senescence pathways were altered at least 2-fold (by increasing or decreasing them) by SU11274 in ovarian cancer cells [41]. These data show its broader action and capability to induce multiple target genes involved in oxidative and metabolic stress [41], so this compound cannot be considered as a c-Met specific inhibitory agent. Crizotinib represents more targeted agent in comparison to SU11274 and it did not alter cellular ATP content in treated cells. Based on the correlation to SU11274-mediated increase in tumor initiation in vivo we concluded that the off-target action of SU11274 is responsible for its protumorigenic action. Furthermore, it favors our hypothesis that melanoma-initiating capability is linked to the metabolic state of cells. The experiments investigating these effects in other tumor types might bring further insight how altering bioenergetic state might potentially support the tumor initiation. SU11274 upregulated almost 2-fold several stem cell markers (Oct3/4, Nanog, AFP and Gata4) in treated cells (Fig. 5a). It also increased activity of RSK1/2/3 kinase based on the phosphotyrosine array analysis. Martin et al. [25] also reported increased ERK activity resulting in RSK1 activation correlating with protumorigenic action in metformin treated melanoma cells A375. However, our data did not confirm any VEGF expression upon SU11274 treatment in contrast to their conclusions that VEGFA upregulation led to protumorigenic action of metformin [25]. It suggested that SU11274 compound exerts its protumorigenic action in the absence of increased VEGF secretion and prompted us to examine bioenergetic regulation.

As recently reviewed, increasing evidence suggests that many types of stem cells rely on anaerobic glycolysis and their stem cell function is regulated by bioenergetic signaling [22, 42]. Similar mechanisms might be operating in cancer stem cells, in fact some studies have already suggested critical role of the metabolic de-regulation for stemness [43]. These findings open novel therapeutic intervention points in cancer. Liu et al. suggested that glycolytic inhibitor 3-BrOP could be combined with standard chemotherapy to target both side population and bulk tumor mass. It was sufficient to treat cells for 24 h with 3-BrOP to achieve antitumor effect in contrast to a platinum-derived agent, which did not affect tumor growth whilst sparing the side population [38]. Higher glycolytic rate seems to be a general characteristic of melanoma cells. Oncogene BRAF, which is constitutively activated also in our melanoma model Rel3 was implicated to be directly involved in reprogramming of cellular metabolism. Dichloroacetate (DCA) as a pyruvate dehydrogenase kinase inhibitor, exerted antimelanoma effect and potentiated its response to specific BRAF inhibition by vemurafenib [21]. Chemosensitivity was not significantly altered in the SU11274-treated cells in vitro and in vivo. We were not able to find any combination of the SU11274 molecule with another chemotherapeutic drug to achieve synthetic lethality (data not shown).

Over the last years, several strategies to target melanoma stem cells were suggested [44, 45]. There were attempts to target a self-renewal pathway of melanoma stem cells thus disabling their ability to replicate as rewieved in [11]. Bioenergetic modulation seems to emerge as novel strategy to target melanoma cancer stem cells. In our work, we present data from the experiments in vivo, which support this hypothesis. Protumorigenic action of small molecule SU11274 could be counteracted by bioenergetic modulator dichloroacetate. On the other hand, glycolytic inhibitor 3-bromopyruvate did not prove suitable, thus showing the specificity in the signaling cascade induced by SU11274 in melanoma cells. More importantly, we observed no antitumorigenic action when dacarbazine was used. Our study further underlines the importance of drug testing in non-adherent spheroid cultures as these might better reflect the efficiency against tumor initiating cells [46].

## Conclusions

Our work highlights a role of bioenergetic modulation in melanoma initiation. It shows that antiproliferative effect in vitro can actually lead to increased tumorigenicity in vivo. In summary, melanosphere cultures were substantially enriched for melanoma-initiating cells in vivo in the absence of any alteration in cancer stem cell markers. Small molecule SU11274 originally intended as a specific c-Met inhibitor significantly reduces melanosphere proliferation, but increases intracellular ATP content, which correlates with an increased tumorigenicity. Tumorigenicity could be reduced in SU11274-treated cells by bioenergetic modulator DCA indicating that glycolytic inhibition could counteract SU11274 mediated effect on melanoma initiation.

### Ethics approval

Animal experiments were performed in the approved animal facility (licence number SK PC 14011) as approved by the institutional ethic committee and by the national competence authority (State Veterinary and Food Administration of the Slovak Republic, registration number Ro 3108/14-221) in compliance with the Directive 2010/63/EU of the European Parliament and the European Council and the Regulation 377/2012 on the protection of animals used for scientific purposes.

### Consent for publication

Not applicable.

### Availability of data and materials

The dataset supporting the conclusions of this article is available at request from the corresponding author.

## Abbreviations

3-BrOP: 3-bromo-2-oxopropionate-1-propyl ester
3BrPA: 3-bromo-pyruvate
bFGF: basic fibroblast growth factor
BrdU: 5-bromo-2-deoxyuridine
DACA: dacarbazine
DCA: dichloroacetate
EGF: epidermal growth factor
EGFP: enhanced green fluorescent protein
ELDA: extreme limiting dilution assay
HGF: hepatocyte growth factor
VEGF: vascular endothelial growth factor
